# SNX10 promotes phagosome maturation in macrophages and protects mice against *Listeria monocytogenes* infection

**DOI:** 10.18632/oncotarget.19644

**Published:** 2017-07-27

**Authors:** Jun Lou, Xiawei Li, Wei Huang, Jingjing Liang, Mingzhu Zheng, Ting Xu, Jun Lyu, Dan Li, Qin Xu, Xuexiao Jin, Guotong Fu, Di Wang, Linrong Lu

**Affiliations:** ^1^ Institute of Immunology, Zhejiang University School of Medicine, Hangzhou, China; ^2^ Program in Molecular and Cellular Biology, Zhejiang University School of Medicine, Hangzhou, China

**Keywords:** SNX10, macrophage, Listeria monocytogenes, phagosome, innate immunity, Immunology and Microbiology Section, Immune response, Immunity

## Abstract

*Listeria monocytogenes* (*L. monocytogenes*), which is a facultative intracellular bacterial pathogen that causes listeriosis, is widely used to study the mammalian immune response to infection. After phagocytosis by professional phagocytes, *L. monocytogenes* is initially contained within phagosomes, which mature into phagolysosomes, where the bacteria are degraded. Although phagocytosis and subsequent phagosome maturation is essential for the clearance of infectious microbial pathogens, the underlying regulatory mechanisms are still unclear. SNX10 (Sorting nexin 10) has the simplest structure of the SNX family and has been reported to regulate endosomal morphology, which might be crucial for macrophage function, including phagocytosis and digestion of pathogens, inflammatory response, and antigen presentation. Our results showed that SNX10 expression was upregulated following *L. monocytogenes* infection in macrophages. It was also revealed that SNX10 promoted phagosome maturation by recruiting the Mon1-Ccz1 complex to endosomes and phagosomes. As a result, SNX10 deficiency decreased the bacterial killing ability of macrophages, and SNX10-deficient mice showed increased susceptibility to *L. monocytogenes* infection *in vivo*. Thus, this study revealed an essential role of SNX10 in controlling bacterial infection.

## INTRODUCTION

Macrophages are important immune cells of the innate immune system that participate in the host defense against bacterial pathogens. They mediate bacterial clearance by internalizing bacteria into phagosomes, which ultimately fuse with lysosomes to kill bacteria [1]. *Listeria monocytogenes* is a food-borne Gram-positive pathogen that can cause listeriosis, such as septicemia and meningitis, particularly in immunocompromised individuals. Following ingestion of various contaminated food products, *L. monocytogenes* crosses the intestinal barrier by invading the intestinal epithelium and reaches internal organs, such as the liver and spleen, *via* the lymphoid system and blood circulation [2, 3]. *L. monocytogenes* can invade both phagocytic and nonphagocytic cells, rapidly replicate within the cytosol and induce cell death. After the bacteria enter host cells, *L. monocytogenes* can escape from these compartments prior to lysosomal fusion to avoid being killed in phagosomes. However, activated macrophages kill *L. monocytogenes* by blocking this phagosomal escape [4]. Therefore, phagosome maturation may affect the vacuolar escape of *L. monocytogenes*: slower maturation will allow more time for *L. monocytogenes* to escape.

Phagosome maturation in macrophages is dependent on the endosomal pathway and is a highly ordered, sequential process. The newly formed phagosome, with no killing ability, is continuously remodeled through fusion with early endosomes, late endosomes and finally lysosomes. These phagosome-endosome interactions are classically viewed as being essential to deliver degradative enzymes to break down the lumenal cargo into peptides, nucleotides and metabolites [5]. Unsurprisingly, various regulators of the endosomal pathway are also required for phagosome maturation, including Rab (Ras-related in brain) GTPases, phosphatidylinositides, CORVET/HOPS tethering complex, SNAREs, and SNXs. The small GTPases Rab5 and Rab7 are key determinants of early and late endosomes, organizing effector proteins into specific membrane subdomains. During endosome maturation, the transition between early and late endosomes is mediated by Rab conversion from Rab5 to Rab7 [6]. Recent studies in *C. elegans* and mammalian cells have shown that the SAND-1/Mon1-Ccz-1 complex promotes Rab conversion by displacing Rab5 from early endosomes and promoting recruitment of Rab7 to late endosomes during endosome maturation and phagosome maturation [7].

Sorting nexins, which were identified as PX domain-containing proteins, have been widely reported to be involved in intracellular protein sorting, trafficking and signal transduction [8]. Recent studies have shown that SNX10 overexpression could induce giant vacuoles in mammalian cells, but the underlying molecular mechanism is still unknown [9]. As the simplest member of the SNX family, SNX10 only contains the PX domain, which binds to various phosphorylated phosphoinositides (PIs) and targets host proteins to cell membranes [10]. The PX domain of SNX10, which specifically binds to PtdIns(3)P, is required for its endosomal localization and vacuolization activity. Moreover, studies have shown that SNX10 is associated with human osteoporosis [11]. SNX10 deficiency impairs osteoclast maturation and bone resorption function. Osteoclasts and macrophages are derived from a common myeloid precursor [12]. Considering the function of SNX10 in maintaining homeostasis of endosomes and vesicles trafficking in osteoclasts, we hypothesized that SNX10 plays a role in the bactericidal activity of macrophages in innate immunity. In this study, our results showed that SNX10 promoted phagosome maturation by mediating Mon1-Ccz1 complex transport in macrophages and thus protected mice against *L. monocytogenes* infection.

## RESULTS

### Bacterial infection upregulates SNX10 expression in mouse macrophages

**Figure 1:**
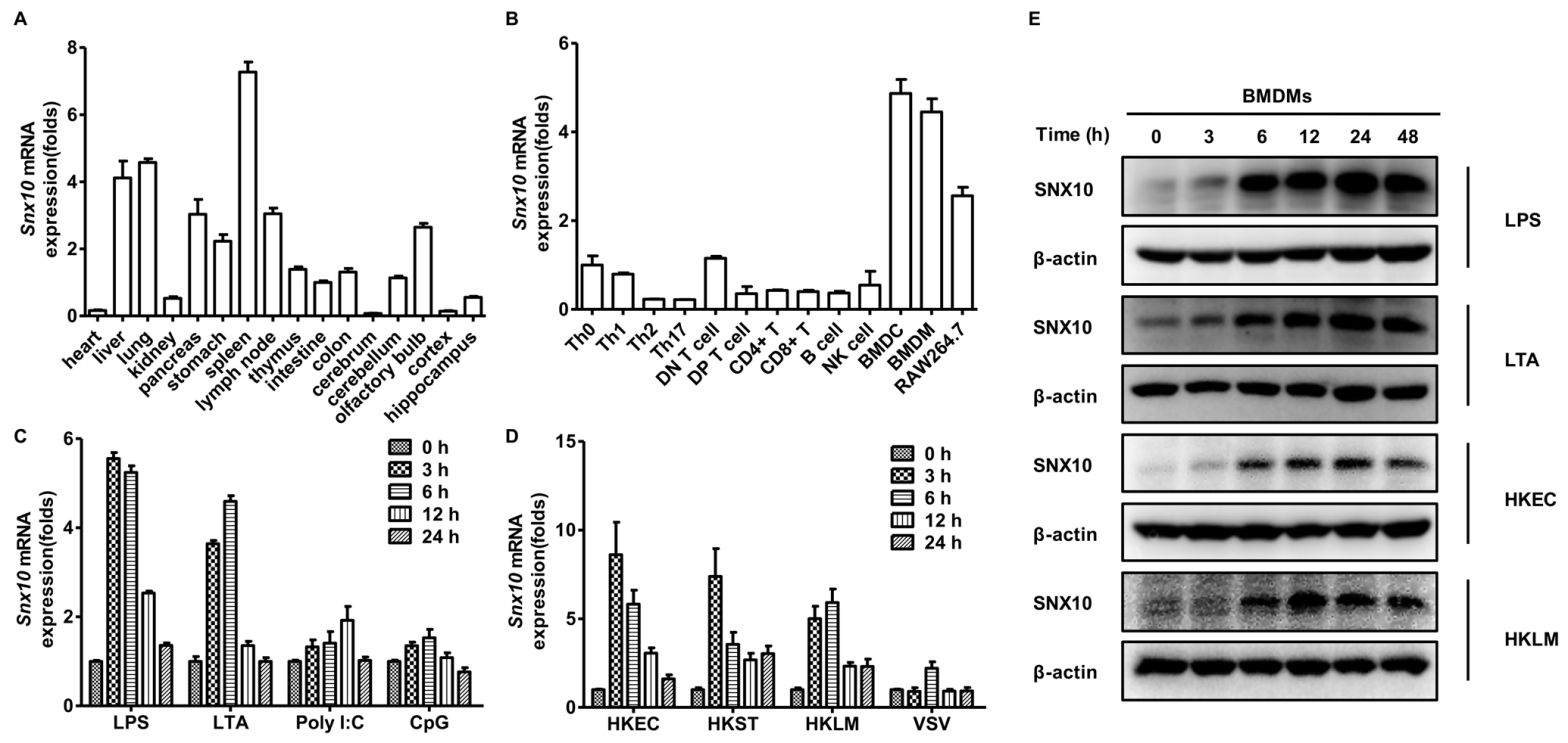
Expression of SNX10 in mouse tissues and cells **A**., **B**. Q-PCR analysis of *Snx10* expression in various mouse tissues **A**. and cells **B**.. **C**., **D**. Q-PCR analysis of *Snx10* expression in BMDMs stimulated with LPS (100 ng/mL), LTA (2 μg/mL), Poly I:C (20 μg/mL), or CpG (0.3 μM) **C**. or infected with HKLM, HKEC, or HKST at a MOI of 20 or VSV at MOI of 1 **D**. for the indicated times. Data were normalized to the expression of the *GAPDH* gene. **E**. Immunoblot analysis of SNX10 protein levels in lysates of BMDMs stimulated with LPS (100 ng/mL) or LTA (2 μg/mL), or infected with HKEC, HKLM at a MOI of 20 for the indicated times. β-actin was used as a loading control. Data are representative of three independent experiments with similar results.

Since gene expression patterns often indicate the functions of their protein products, we first investigated the murine tissue distribution of SNX10 mRNA by Q-PCR. Our results showed that SNX10 mRNA was highly expressed in spleen, liver and lung (Figure 1A). Next, we investigated the expression of SNX10 in different types of immune cells. We found that SNX10 was highly expressed in BMDMs and bone marrow-derived dendritic cells (Figure 1B). Macrophages and dendritic cells are the frontline cells of the innate immune system. They sense and immediately respond to invading pathogens, thus providing an early defense against external attack [13]. Toll-like receptors (TLRs) are a family of pattern recognition receptors (PRRs) that function as primary sensors of the innate immune system to recognize microbial pathogens. Ligands binding to TLRs invoke a cascade of intracellular signaling pathways that induce the production of factors involved in inflammation and immunity [14]. To investigate the function of SNX10 in macrophages, we examined its expression in BMDMs after stimulation with different TLR ligands. Upon stimulation with LPS (TLR4 ligand) and LTA (TLR2 ligand), *Snx10* expression was significantly increased; in contrast, *Snx10* expression was not significantly altered following stimulation with Poly I:C (TLR3 ligand) and CpG (TLR9 ligand) (Figure 1C). LPS is a major surface membrane component in almost all Gram-negative bacteria, and LTA is a major constituent of the cell wall of Gram-positive bacteria. Next, we detected the expression of SNX10 in BMDMs after infection with Gram-negative bacteria (*E. coli* and *Salmonella typhimurium*) and Gram-positive bacteria (*L. monocytogenes*). Similar results were observed in both types of bacterial infection; the mRNA expression of *Snx10* in BMDMs was significantly upregulated after infection with heat-killed *L. monocytogenes* (HKLM), heat-killed *E. coli* (HKEC) and heat-killed *S. typhimurium* (HKST) but SNX10 upregulation after infection with VSV(vesicular stomatitis virus) was negligible (Figure 1D). Immunoblot analysis also showed that the protein levels of SNX10 in primary BMDMs substantially increased after LPS, LTA, or bacterial challenge (Figure 1E). These results suggested a possible role of SNX10 in the anti-bacterial response of macrophages.

### SNX10 promotes bactericidal activity in macrophages

Cytokines produced by macrophages are important mediators of immune responses. When macrophages are exposed to inflammatory stimuli, they secrete cytokines such as tumor necrosis factor (TNF), IL-6, and IL-12 [15]. Recent evidence indicates that *L. monocytogenes* has been recognized by TLR2, NOD1 and NOD2, resulting in the activation of mitogen-activated protein kinases (MAPK), NF-κB pathways, and pro-inflammatory gene expression [16, 17]. To determine whether SNX10 contributes to the regulation of TLR signal pathways and the production of inflammatory cytokines upon *L. monocytogenes* infection, we cultured BMDMs from WT and SNX10-deficient mice *in vitro*. Upon stimulation with LTA or infection with HKLM, surprisingly, there was no significant difference in the production of TNF-α, IL-6 and IL-12p40 between *Snx10*-/- and WT BMDMs in response to LTA and HKLM (Supplementary Figure 1A and 1B). Moreover, SNX10 deletion did not alter the phosphorylation of p38, Jnk, Erk and the NF-κB p65 subunit (Supplementary Figure 1C and 1D). These results suggested that loss of SNX10 does not affect cytokine production in macrophages after *L. monocytogenes* infection *in vitro*.

**Figure 2:**
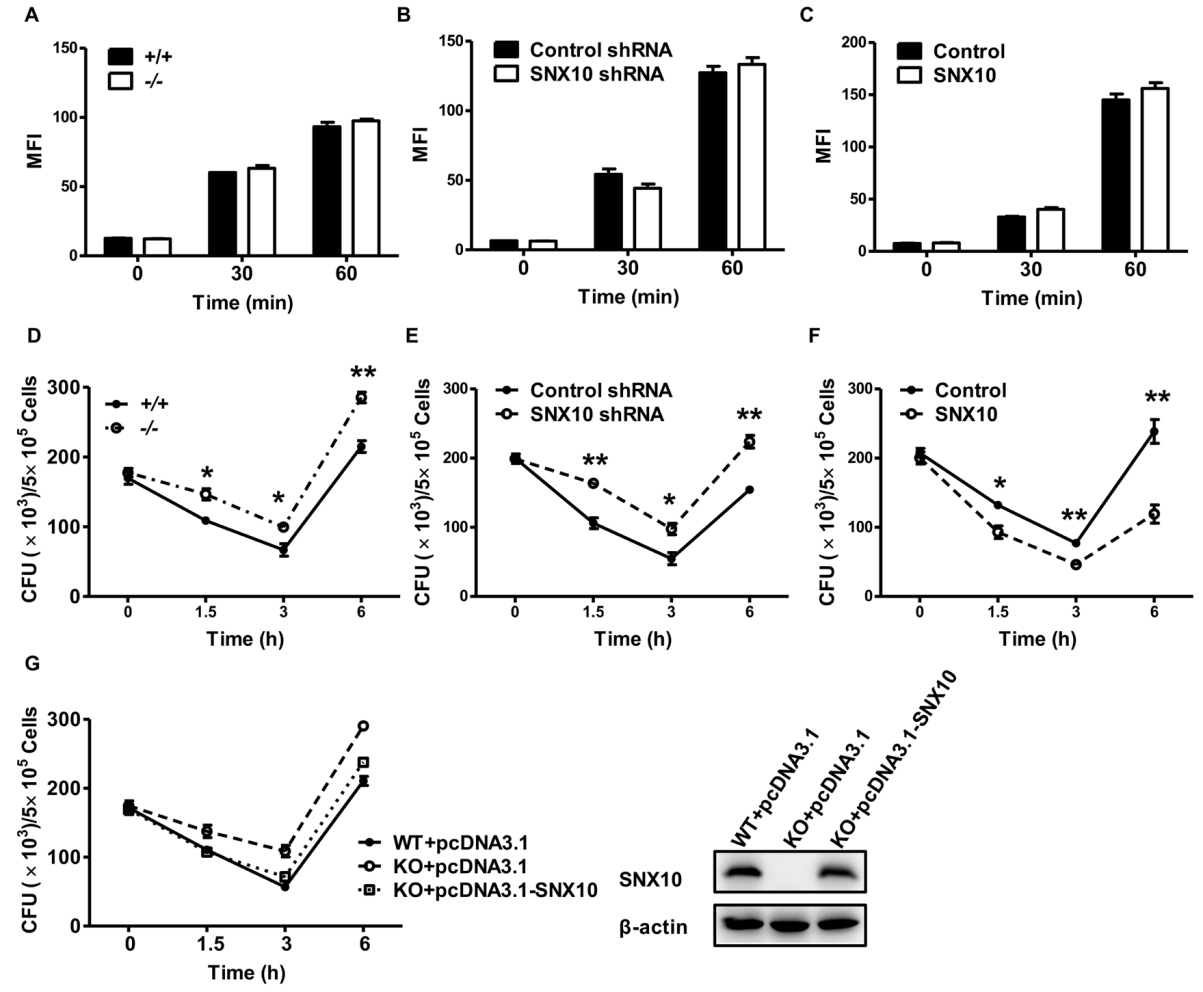
SNX10 regulates bactericidal activity in macrophages **A**.-**C**. Flow cytometry analysis of WT and *Snx10*-/- BMDMs **A**., or RAW264.7 cells stably expressing control and SNX10 shRNA **B**., or stably expressing control and SNX10 **C**. infected for the indicated times at 37°C with Alexa Fluor 488-labeled *L. monocytogenes* at an MOI of 10, data are expressed as the MFI of the macrophage populations. **D**.-**F**. Pathogen burden in WT and *Snx10*-/- BMDMs **D**. or RAW264.7 cells stably expressing control and SNX10 shRNA **E**. or stably expressing control and SNX10 **F**. infected with *L. monocytogenes* (MOI, 5) for the indicated time; data are expressed as CFUs. *P < 0.05, **P < 0.01. **G**. Left, pathogen burden in WT and *Snx10*-/- BMDMs transfected with the pcDNA3.1 or pcDNA3.1-SNX10 for 36 h and then infected with *L. monocytogenes* (MOI, 5) for the indicated time; Right, immunoblot analysis of SNX10 protein levels in lysates of BMDMs were transfected with the pcDNA3.1 or pcDNA3.1-SNX10 by using Lipofectamine LTX for 36 h, β-actin was used as a loading control. Data are representative of three independent experiments with similar results.

Phagocytic and microbial killing ability are also hallmark functions of macrophages in innate immunity [18]. To investigate the function of SNX10 in phagocytosis, we incubated Alexa Fluor 488-labeled *L. monocytogenes* with macrophage *in vitro*. Flow cytometric analysis showed that there was no difference in MFI between WT and *Snx10*-/- BMDMs at 30 and 60 min postinfection, suggesting that the loss of SNX10 did not affect the uptake of *L. monocytogenes* by macrophages (Figure 2A). Furthermore, we also stably transfected RAW264.7 cells with SNX10 shRNA or Flag-SNX10 by lentivirus (Supplementary Figure 2A and 2B). And we found that either knockdown or overexpression of SNX10 did not affect the growth (Supplementary Figure 2C and 2D) or phagocytosis (Figure 2B and 2C) of macrophages. To determine whether macrophages from *Snx10*-/- mice have an innate defect in their ability to kill *L. monocytogenes*, we incubated BMDMs from WT and *Snx10*-/- mice with *L. monocytogenes* at a MOI of 5 for 0, 1.5, 3 and 6 h. Notably, *Snx10*-/- BMDMs exhibited impaired bacterial clearance. The quantity of live *L. monocytogenes* in *Snx10*-/- BMDMs was greater than that in WT cells at 1.5, 3 and 6 h postinfection (Figure 2D). Similar results were observed in *Snx10* knockdown and control macrophages (Figure 2E). On the contrary, there were substantially fewer live *L. monocytogenes* in SNX10-overexpressing macrophages compared to those in control cells at 1.5, 3, 6 h postinfection (Figure 2F). More importantly, our results also showed that re-expression of SNX10 in *Snx10*^-/-^ BMDMs regained the cells’ ability to kill *L. monocytogenes* to a similar level to the WT cells (Figure 2G). These data suggested that SNX10 did not affect the uptake of *L. monocytogenes* but enhanced bactericidal activity of the macrophages.

### SNX10 promotes the maturation of *L. monocytogenes*-containing phagosomes

**Figure 3:**
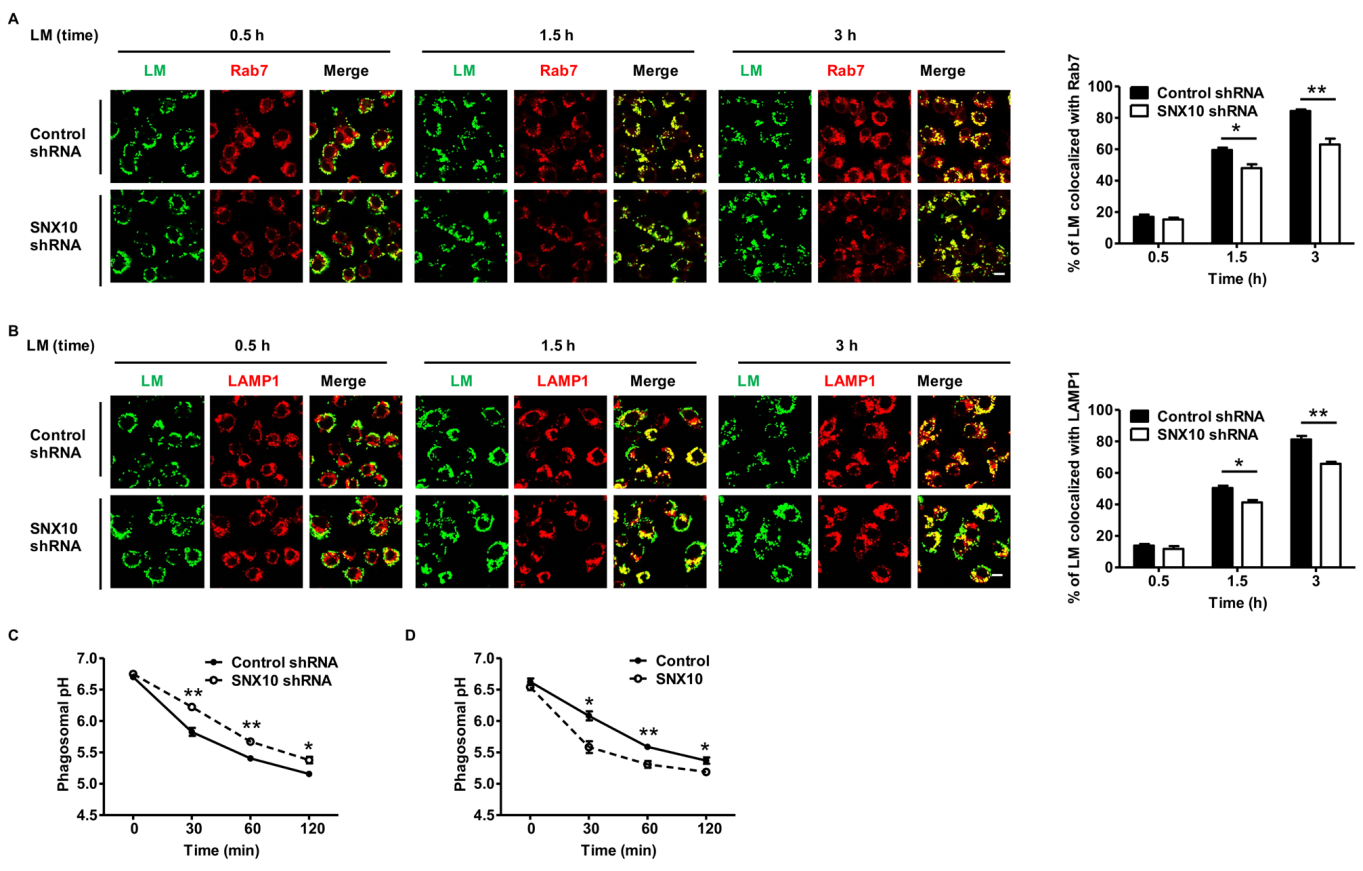
SNX10 promotes the maturation of *L. monocytogenes*-containing phagosomes **A**., **B**. Confocal microscopy of RAW264.7 cells stably expressing control or SNX10 shRNA infected with heat-killed *L. monocytogenes* (MOI, 20) for the indicated time. The images (left) showed the co-localization of heat-killed Alexa Fluor 488-*L. monocytogenes* with Rab7 **A**., or LAMP1 **B**.. Green, Alexa Fluor 488-*L. monocytogenes* (LM); Red, Rab7 or LAMP1. Co-localization of the two colors is depicted by yellow in the images. Scale bars represent 10 μm. The percentage of co-localization (right) between *L. monocytogenes* and Rab7 or LAMP1 was measured as described in Materials and Methods. **C**., **D**. Kinetics of acidification of heat-killed *L. monocytogenes*-containing phagosomes in RAW264.7 cells stably expressing control or SNX10 shRNA **C**., and control or SNX10 **D**.. *P < 0.05, **P < 0.01. Data are representative of three independent experiments with similar results.

Considering that SNX10 promotes bactericidal activity in macrophages, we wonder whether SNX10 results in an alteration in the endocytosis pathway during bacterial infection. To elucidate the degradation process of bacteria, researchers have studied a number of different marker proteins (e.g., Rab7, LAMP1) involved in phagosomal maturation on the surface of bacteria-containing phagosome [19]. We first examined the co-localization of *L. monocytogenes* with Rab7, a marker of late endosomes and phagosomes, in both control cells and SNX10 knockdown cells after infection. The results showed that the percentage of co-localization of *L. monocytogenes* with Rab7 increased from 0.5 to 3 h after infection. More importantly, at both 1.5 and 3 h, the percentages of *L. monocytogenes* co-localized with Rab7 were significantly decreased in SNX10 knockdown cells (Figure 3A). Similar results were obtained in the co-localization of *L. monocytogenes* with LAMP1 (Figure 3B), a trans-membrane glycoprotein of late endosomes and lysosomes.

Considering that phagosomes acquire various hydrolases and undergo a progressive acidification during the course of maturation, we investigated whether SNX10 promotes phagosomal acidification upon bacterial infection [20]. We used FACS to analyze the pH of *L. monocytogenes*-containing phagosomes. Heat-killed bacteria were dually labeled with the pH-sensitive FITC and the pH-insensitive Alexa Fluor 647 while undergoing phagocytosis by macrophages [21]. We found that the *L. monocytogenes*-containing phagosomes acidified more rapidly in control macrophages than that in SNX10 knockdown macrophages (Figure 3C). In contrast, *L. monocytogenes*-containing phagosomes acidified more rapidly in SNX10 overexpression macrophages than that in control macrophages (Figure 3D). These data suggested that SNX10 promoted the maturation of *L. monocytogenes*-containing phagosomes.

### SNX10 promotes late endosome and late phagosome maturation in macrophage

**Figure 4:**
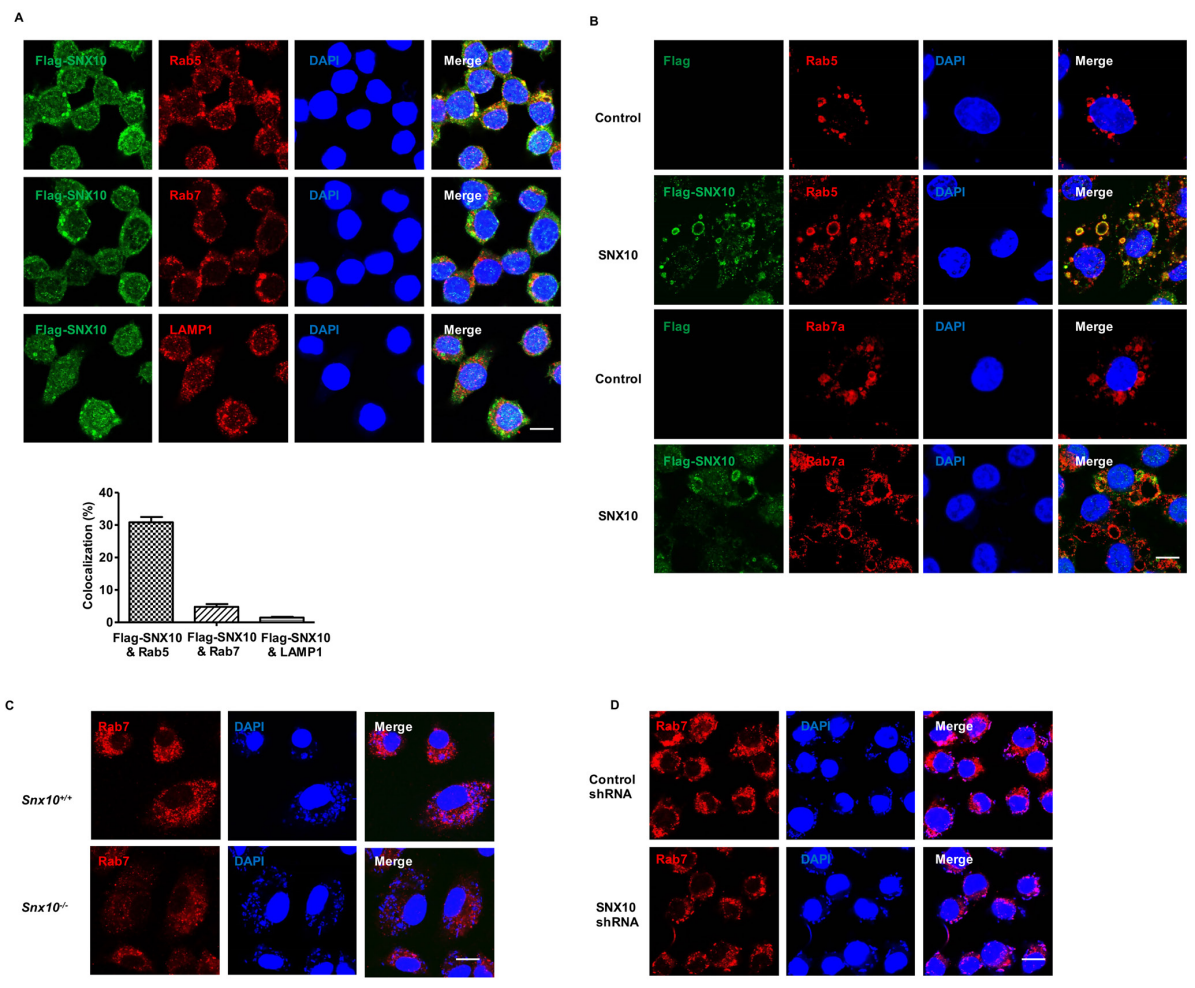
SNX10 promotes late endosome and late phagosome maturation in macrophage **A**. Confocal images showing the co-localization of Flag-SNX10 (Green) with Rab5, Rab7 or LAMP1 (Red) in RAW264.7 cells line stably expressing Flag-SNX10. Bottom, The percentage of co-localization between Flag-SNX10 and Rab5, Rab7 or LAMP1 was measured as described in Materials and Methods. **B**. Confocal images showing the co-localization of Flag-SNX10 (Green) with Rab5, Rab7 (Red) in RAW264.7 cells line stably expressing control or Flag-SNX10 under LPS (1 μg/mL) stimulation for 24 h. Co-localzation of the two colors is depicted by yellow in the images. **C**., **D**. Confocal images showed the late endosomes or phagosomes (Rab7-positive) in WT and *Snx10*-/- BMDMs **C**. or RAW264.7 cells stably expressing control or SNX10 shRNA **D**. after infection with *L. monocytogenes* (MOI, 20) for 3 h. Scale bars represent 10 μm. Data are representative of three independent experiments with similar results.

Previous studies have reported that SNX10 induced giant vacuoles in mammalian cells. These giant vacuoles were Rab7-positive organelles, suggesting a possible role of SNX10 in promoting the process of the early endosome converting to late endosome during endosome maturation. To determine whether SNX10 has a similar function in macrophage, we first examined the subcellular localization of SNX10 and found that SNX10 was mainly located in early endosomes (Rab5-positive organelles) (Figure 4A). Notably, SNX10 was predominantly recruited to early endosomes during the activation of macrophages by LPS or LTA stimulation, but not late endosomes (Supplementary Figure 3A). Our results also showed that SNX10 overexpression could induce giant vacuoles in LPS-stimulated macrophages, which consisted of a large number of late endosomes but only a small number of early endosomes (Figure 4B). These results were consistent with the previously reported results, and suggesting that SNX10 could also regulate the process of early endosomes converting to late endosomes in macrophages. Phagosome maturation is closely related to endosome maturation. New phagosomes merge with early endosomes to become early phagosomes and in turn the early phagosomes then merge with late endosomes to become late phagosomes. Finally, late phagosomes merge with lysosomes to become the ultimate degradative organelles, phagolysosome [22]. During *L. monocytogenes* infection, SNX10 was also recruited to early endosomes (Rab5-positive organelles) (Supplementary Figure 3B). More importantly, SNX10 deficiency led to a reduction of late endosomes and phagosomes (Rab7-positive organelles) during *L. monocytogenes* infection in BMDM (Figure 4C), but not evident in early endosomes and phagosomes (Rab5-positive organelles) (Supplementary Figure 3C). Similar results were observed in *Snx10* knockdown and control macrophages (Figure 4D and Supplementary Figure 3D). These data suggested an essential role of SNX10 in the process of early endosome converting to late endosome and promoted late phagosome maturation in macrophages.

### SNX10 promotes the recruitment of the Mon1-Ccz1 complex to phagosomes and endosomes

**Figure 5:**
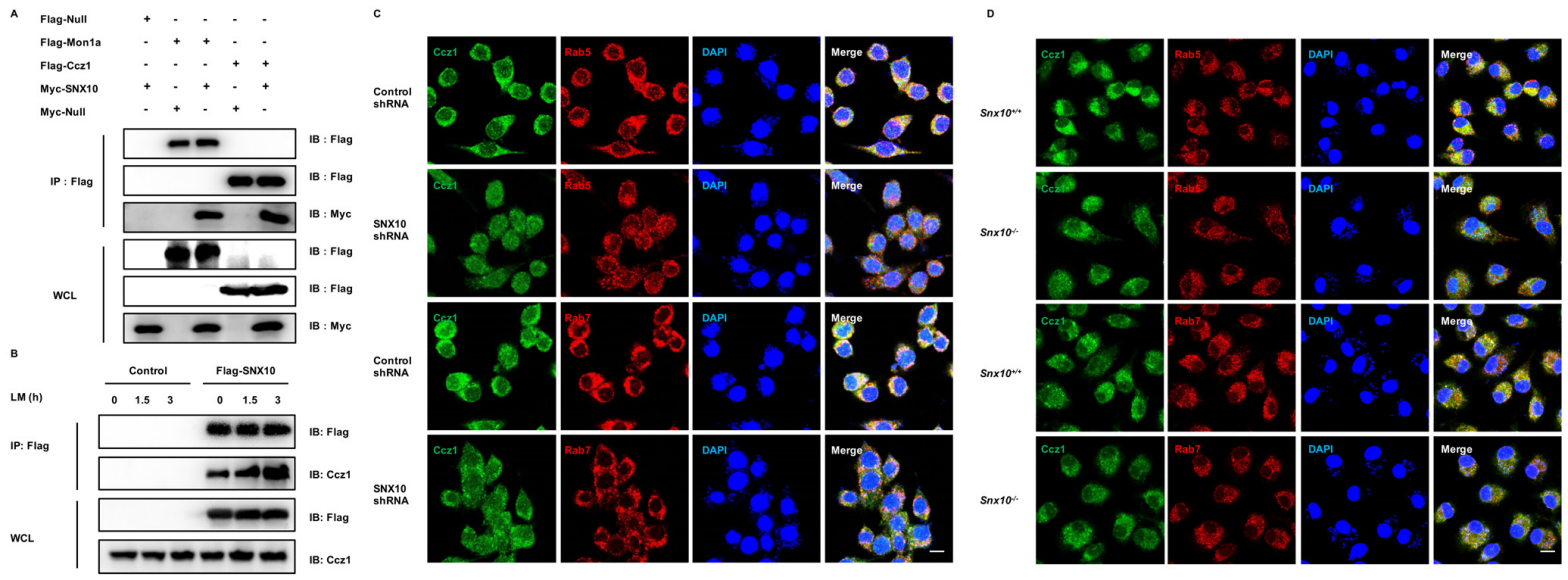
SNX10 recruits the Mon1-Ccz1 complex to endosomes **A**. Co-immunoprecipitation of the interactions between SNX10 and Mon1a or Ccz1 in 293T cells. **B**. Co-immunoprecipitation of the interactions between SNX10 and Ccz1 in RAW264.7 cells stably expressing control or Flag-SNX10 after *L. monocytogenes* (MOI, 10) infection for the indicated time. **C**., **D**. Confocal images showing the co-localization of Ccz1 (Green) with Rab5 or Rab7 (Red) after *L. monocytogenes* (MOI, 10) infection for 3 h in RAW264.7 cells stably expressing control or SNX10 shRNA **C**., or in WT and *Snx10*-/- BMDMs **D**.. Co-localization of the two colors is depicted by yellow in the merged images. Scale bars represent 10 μm. Data are representative of three independent experiments with similar results.

Given the function of SNX10 in the maturation of late endosomes and phagosomes, various protein complexes involved in promoting endosome and phagosome maturation were screened (data not show), and we found that SNX10 interacted with the Mon1-Ccz1 complex in 293T cells (Figure 5A). Further results showed that SNX10 could also bind with CCZ1 in macrophages and notably bind more CCZ1 following *L. monocytogenes* infection (Figure 5B). The Mon1-Ccz1 complex is the guanine nucleotide exchange factor (GEF) for Rab7 and is required for endosomal maturation and fusion at the vacuole/lysosome. During endosome maturation, it could be recruited from the cytosol to endosomal membranes and functions as a switch that inhibits the recruitment of Rab5 and facilitates the activation of Rab7 [23]. Our results also showed that SNX10 deficiency in RAW264.7 (Figure 5C) or BMDM (Figure 5D) inhibited the co-localization of Ccz1 with Rab5 or Rab7 upon *L. monocytogenes* infection, suggesting that the recruitment of the Mon1-Ccz1 complex to early and late endosomes was impaired in macrophages. These data revealed that SNX10 could promote the recruitment of the Mon1-Ccz1 complex to early endosomes and phagosomes and the maturation of phagosome during bacterial infection.

### SNX10-deficient mice show increased susceptibility to *L. monocytogenes* infection

**Figure 6:**
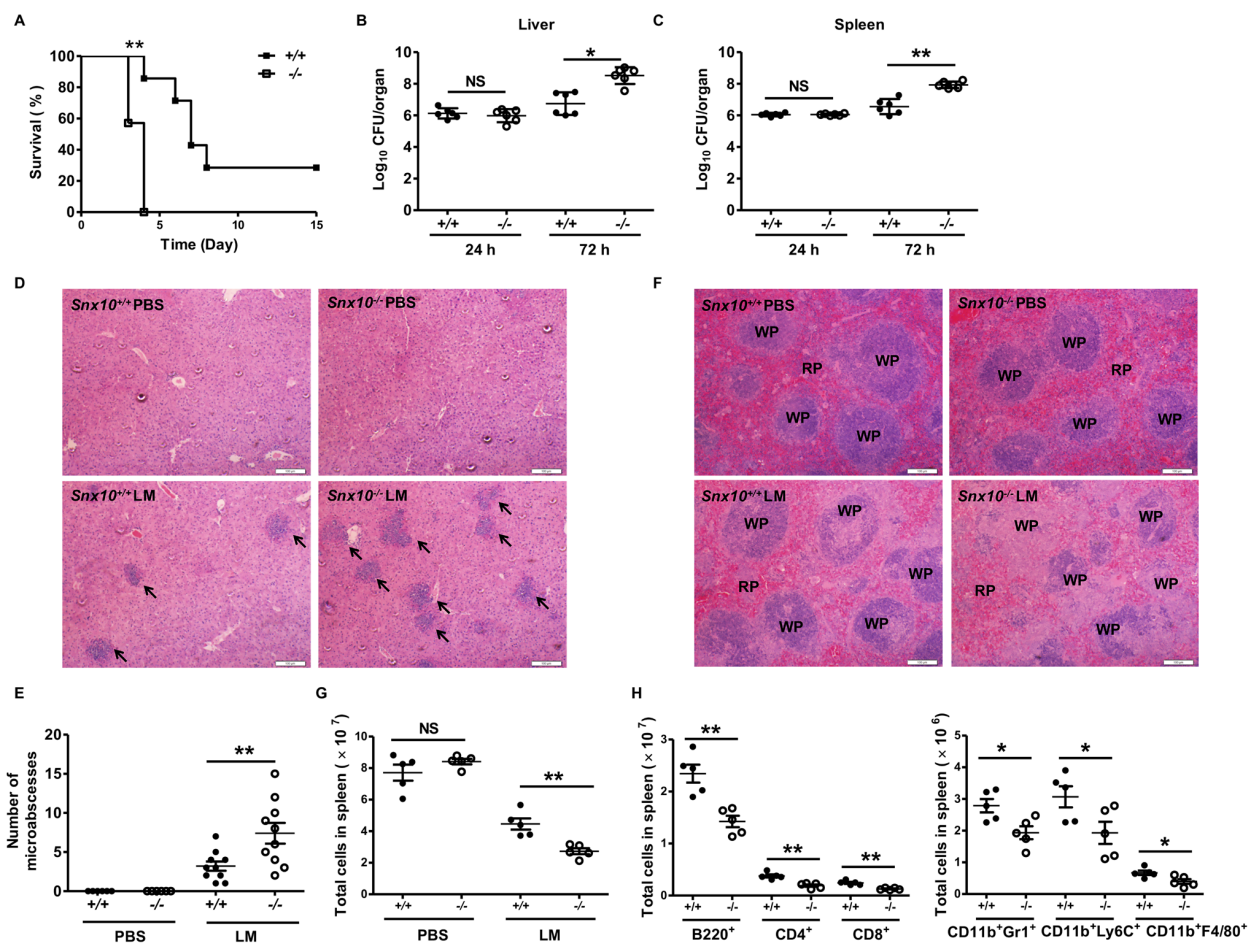
SNX10-deficient mice are more susceptible to bacterial sepsis **A**. Survival of WT and *Snx10*-/- mice after intravenous injection with 2×10^5^ *L. monocytogenes* (LM). Mice were monitored every day after injection. *n* = 7 mice per genotype. *P* < 0.01 (log-rank test). **B**., **C**. Bacterial load in the liver **B**. or spleen **C**. assessed 24 or 72 h after infection as in A. **D**. Microscopic images of H&E staining of the liver after infection WT and *Snx10*-/- mice 72 h after intravenous injection with PBS or *L. monocytogenes* as A, Arrows indicate the microabscesses. **E**. The quantitative analysis of microabscesses in D. The data were quantitated from two sections per mouse in five mice per group. **F**. Microscopic images of H&E staining of spleen from WT and *Snx10*-/- mice 72 h after intravenous injection with PBS or *L. monocytogenes* as A. Sections are representative of at least five mice per group; scale bars represent 100 μm. RP, red pulp; WP, white pulp. **G**. Total splenocytes were counted in WT and *Snx10*-/- mice 72 h after intravenous injection with PBS or *L. monocytogenes* as A. **H**. Different cell types were counted in WT and *Snx10*-/- mice 72 h after intravenous injection with *L. monocytogenes* as A. NS, no significance. *P < 0.05, **P < 0.01. Data are representative of three independent experiments with similar results.

We next examined the function of SNX10 *in vivo* by injecting wild-type (WT) and *Snx10*-/- mice with *L. monocytogenes* and monitoring their survival. We found that SNX10-deficient mice were more susceptible to *L. monocytogenes* infection than WT mice (Figure 6A). When systemic infection occurs, *L. monocytogenes* enter the circulation and are transported to the liver and spleen, where they infect hepatocytes and macrophages [24]. To determine whether SNX10 controls *L. monocytogenes in vivo*, we investigated the bacterial burden of liver and spleen from WT and *Snx10*-/- mice at 24 and 72 h after infection. At 24 h, no significant difference in bacterial burden was observed in the spleen and liver between the two genotypes. However, at 72 h, a significant difference in CFUs was observed in both organs. *Snx10*-/- mice had more bacteria in their liver and spleen than WT mice (Figure 6B and 6C).

Microabscess formation is a histological hallmark of *L. monocytogenes*-infected livers [25]. To evaluate the microabscess formation in the liver of *L. monocytogenes*-infected WT and *Snx10*-/- mice, we stained the liver at 72 h postinfection with H&E and found that there were more microabscesses in *Snx10*-/- mice compared with WT mice (Figure 6D and 6E). To further investigate the susceptibility of *Snx10*-/- mice to *L. monocytogenes*, we examined the spleen histology of infected WT and *Snx10*-/- mice. As shown by H&E staining, the cellular destruction observed in the white pulp of the spleen of *Snx10*-/- mice was substantially more severe than that in the WT mouse spleen (Figure 6F). Previous reports suggested that *L. monocytogenes* infection induces lymphocyte and myeloid cell death [26]. To quantify the cell populations in the spleen of WT and *Snx10*-/- mice, we harvested and quantified splenocytes on day 3 postinfection. On day 3, there was no significant difference between WT and *Snx10*-/- mice after injection with PBS, but *Snx10*-/- mice had fewer splenocytes than WT mice after injection with *L. monocytogenes* (Figure 6G). The spleen consists of many types of immune cells that play different roles in the course of an infection. We further counted the number of different cell types in WT and *Snx10*-/- mice. There were similar numbers of CD4^+^ and CD8^+^ T cells, B cells (B220^+^), neutrophils (CD11b^+^Gr1^+^), inflammatory monocytes (CD11b^+^Ly6C^+^), and macrophages (CD11b^+^F4/80^+^) observed in WT and *Snx10*-/- mice after injection with PBS (Supplementary Figure 4). However, the number of every cell type examined was significantly fewer in *Snx10*-/- mice than in their WT counterparts during *L. monocytogenes* infection (Figure 6H). These results revealed that the loss of SNX10 did not affect the maturation of immune cells, but instead led to faster replication of *L. monocytogenes* in the spleen and thus induced more serious cell death and tissue damage.

Given that cytokine storm is also an important factor for sepsis and even death *in vivo* [27, 28], we therefore examined serum cytokine levels in WT and *Snx10*-/- mice at 24 an 48 h after *L. monocytogenes* infection. Our result show that there was no difference in the serum levels of IFN-γ, TNF-α, IL-6 or IL12p40 between WT and *Snx10*-/- mice at both time point (Supplementary Figure 5A). We also challenged mice with LPS and found that LPS injection led to similar death rates (Supplementary Figure 5B) and cytokine production levels (Supplementary Figure 5C) in both strains. These findings suggested that the susceptibility of *Snx10*-/- mice to *L. monocytogenes* infection was not due to changes in cytokines production but the weakened bacterial clearing ability in response to bacterial infection *in vivo*.

## DISCUSSION

Sorting nexins are a large group of proteins that are localized in the cytoplasm and have the potential for receptor degradation and membrane traffic in the cell. But the functions of most of the family in the immune system are still unknown. Previous studies have demonstrated that SNX10 regulate endosomal morphology in mammalian cells, suggesting a possible role of SNX10 in regulating endocytosis pathway during bacterial infection in macrophage. In our study, we found that SNX10 was highly expressed in mouse macrophages and was upregulated upon bacterial infection, which suggested a possible role of SNX10 in the anti-bacterial response of macrophages.

Phagocytosis by macrophages for ingestion and degradation of microbes is not only the first line of defense in the innate immune response to infection but is also important for antigen processing and presentation, which helps to drive the adaptive immune response. Although some SNX family proteins, such as SNX3 and SNX5 [29, 30], have been shown to affect phagocytosis, our results suggested that SNX10 deficiency did not affect the phagocytosis of macrophages following bacterial infection. However, loss of SNX10 decreased the efficiency of killing *L. monocytogenes*. Phagosomes mediate pathogen killing in macrophages. Previous studies have suggested that SNX3 is a component of a tubular endosomal network induced by *Salmonella* infection and is involved in the maturation of *Salmonella*-containing vacuoles [31]. Vacuolar escape of *L. monocytogenes* could be inhibited by maturation of vacuoles to lysosome-like compartments. Slower vacuolar maturation may be more permissive for efficient vacuolar escape of *L. monocytogenes* for multiple reasons. Although many endosome components in vesicular trafficking have been shown to prevent efficient escape of *L. monocytogenes* in human cells [32], our results demonstrated that SNX10 is a novel endosomal protein in anti-*L. monocytogenes* infection.

Previous studies have demonstrated that SNX10 induced giant vacuoles in mammalian cells, while Rab7 mutation inhibited this process. However, the underlying mechanism remains unknown. Here in macrophages, we found that SNX10 was predominantly recruited to early endosomes during the activation of macrophages. The overexpression of SNX10 induced the formation of giant late endosomes, and the deficiency of it led to a reduction in late endosomes and phagosomes during bacterial infection. Considering that the process of phagosome and endosomes maturation is very similar and inseparable, we concluded that SNX10 promoted the maturation of both late endosomes and late phagosomes. Furthermore, we found that SNX10 interacted with the Mon1-Ccz1 complex, and loss of SNX10 reduced the recruitment of this complex to phagosomes and endosomes during bacterial infection. The timing of Mon1-Ccz1 complex recruited to early endosomes is critical for Rab conversion. SNX10 interacted with Mon1-Ccz1 complex and then specifically bond to PtdIns(3)P, which might not only stabilized Mon1-Ccz1 complex on membranes, but also stimulated Mon1-Ccz1 GEF activity [33]. SNX10 was shown to be essential for promoting Rab5 to Rab7 conversion, which mediated the conversion of early endosomes and phagosomes to late endosomes and phagosomes. But further studies should be done to investigate into more detailed molecular mechanism.

In addition, SNX10 shares the highest sequence homology with SNX11 among the SNX proteins. Recent studies demonstrated that SNX11 and SNX10 both contain a novel PX domain with two additional α-helices next to the conventional PX domain, named the PX extended (PXe) domain. Thus, SNX11 may inhibit SNX10-induced vacuolization by competitively binding target proteins [34]. Further studies are needed to verify whether SNX11 participates along with SNX10 in bacterial clearance and phagosome maturation.

The phagocytic function of macrophages plays a pivotal role in eliminating invading pathogens in various tissues [35]. *In vivo*, we found that loss of SNX10 in mice resulted in higher mortality, increased bacterial burden, and more serious liver and spleen damage upon *L. monocytogenes* infection. This result further supported that the function of SNX10 in macrophages was important for protecting mice against bacterial infection, although we were not able to rule out the functions of SNX10 in other cells since total knockout mice were used in our study.

To the best of our knowledge, the results of this study showed for the first time that SNX10 has a critical role in providing host protection against *L. monocytogenes* infection, identifying this protein as a potentially important target for future therapeutic strategies against bacterial infection.

## MATERIALS AND METHODS

### Mice

*Snx10*-/- mice (B6.129-*Snx10*) were purchased from Shanghai Model Organisms Center, Inc., and backcrossed for over 5 generations onto the C57BL/6 background. Female mice at 8-12 weeks of age were used for the *in vivo* animal experiments. All mice were housed in the Experimental Animal Center of Zhejiang University and were monitored daily by laboratory and animal facility personnel. The animal experiments were performed in accordance with Zhejiang University institutional guidelines and the protocols were approved by the Ethics Committee of Zhejiang University (ZJU20170800).

### Reagents

Lipopolysaccharide (LPS, *Escherichia coli 0111:B4*), lipoteichoic acid (LTA), and polyinosinic:polycytidylic acid (Poly I:C) were from Sigma-Aldrich, and phosphorothioate-modified CpG ODN was synthesized by Sybersyn. Phospho-p44/42 MAPK (Erk1/2) (Thr202/Tyr204) (197G2) rabbit mAb, Phospho-SAPK/JNK (Thr183/Tyr185) (81e11) rabbit mAb, Phospho-p38 MAPK (Thr180/Tyr182) (D3F9) rabbit mAb, Phospho-NF-κB p65 (Ser536) (93H1) rabbit mAb, EEA1 (C45B10) rabbit mAb, Rab5 (C8B1) rabbit mAb, and Rab7 (D95F2) rabbit mAb were from Cell Signaling Technology. CD107a/LAMP-1 mouse mAb was from BioLegend; β-actin mouse mAb and Ccz1 antibodies (L-20) were obtained from Santa Cruz Biotechnology; SNX10 antibody (HPA015605) was acquired from Sigma-Aldrich. Mouse CD4-FITC, CD8-PE, B220-APC, CD11b-PE, CD11b-APC, Gr1-FITC, Ly6C-PE, and F4/80-APC antibodies were purchased from eBioscience. Fluorescein isothiocyanate isomer I (FITC) and Cell Counting Kit-8 (CCK-8) were from Sigma-Aldrich; Alexa Fluor^®^ 488 dye and Alexa Fluor^®^ 647 dyes were from Thermo Fisher Scientific.

### Plasmid construction

The cDNAs encoding mouse SNX10 were amplified from cDNA of mouse bone marrow-derived macrophages (BMDMs) by PCR and cloned into pcDNA3.1-6×myc. The PCR primers were as follows: SNX10 (Forward: 5′- ATTAGAATTCTATGTTCCCAGAACAGCAGAAAG-3′, Reverse:5′-ATATCGCT CGAGTCAGGACTCCTGCAGAGCTGGGCTCGTT-3′). The cDNAs encoding mouse Mon1a or Ccz1 were amplified from cDNA of mouse BMDMs by PCR and cloned into pCMVtag2C. The PCR primers were as follows: Mon1a (Forward: 5′-CAGGAATTCGAATGGCTGCTGACATGCAGAG-3′, Reverse: 5′-GAGGTCGACTCAATAGGTGAGGGGCGTGAG-3′); Ccz1 (Forward: 5′-ATTAGAATTCGAATGGCGGCAGCCGCGGCCG-3′, Reverse: 5′-CCGCTCGAGGTTCAATCCAAGAAGAAGATG-3′). Short hairpin RNA (shRNA) was cloned into pLVX-shRNA1 (Clontech) for lentivirus packaging. The shRNA target sequences were as follows: SNX10 shRNA, 5′-ACCAGAACTTCCGTCTAAA-3′; Control shRNA, 5′-GCAAGCTGACCCTGAAGTTC-3′. All constructs were confirmed by sequencing.

### Cell culture and stable cell line generation

RAW 264.7 and HEK293 cell lines were purchased from the American Type Culture Collection (ATCC) (Manassas, VA) and cultured in Dulbecco's modified Eagle's medium (DMEM) supplemented with 2 mM glutamine, 100 units/mL penicillin and 100 mg/mL streptomycin sulfate, and 10% heat-inactivated fetal bovine serum (FBS) (Gibco) at 37°C in the presence of 5% CO_2_. For BMDM culture, bone marrow was collected from femurs and tibiae of mice (6-8 weeks of age) and cultured in DMEM supplemented with macrophage colony-stimulating factor (20 ng/mL) in addition to 10% (vol/vol) FBS. Murine BMDM were transfected using Lipofectamine LTX with Plus™ transfection reagent (Invitrogen) according to the manufacturer's instructions.

RAW264.7 cells stably expressing Flag-SNX10 or SNX10 shRNA were generated by lentivirus transduction. Lentivirus was produced by co-transfection of 293T cells with Flag-SNX10 in the pLVX-puro (or shRNA in pLVX-shRNA1), pVSV-G, pLP1 and pLP2 plasmids using Lipofectamine 2000 (Invitrogen). Viral supernatants were harvested at 48-72 h after transfection, were passed through a 0.45-μm filter, diluted 2:3 with fresh medium containing 8 μg/mL polybrene and used to infect the RAW264.7 at 80% confluence. For stably expressing Flag-SNX10 or SNX10 shRNA in macrophages, RAW264.7 cells were selected with the puromycin (5 μg/mL) and were pooled for further experiments.

### RNA isolation and quantitative PCR (Q-PCR)

Total RNA was extracted using RNAiso Plus (TaKaRa), and reverse transcription (RT) was carried out using a PrimeScript™ RT-PCR kit (TaKaRa). Then, Q-PCR was performed using a 96-well CFX-96 detection system (Bio-Rad Laboratories) with SYBR Premix Ex Taq™ (TaKaRa, Cat. No. DRR041A).

The corresponding primers were as follows:

Mouse *SNX10* (Forward: 5′-ATGTTCCCAGAACAGCAGAAAGAG-3′, Reverse: 5′-GTAATTGTACCAGCAACGCGTTGC-3′);

Mouse *GAPDH* (Forward: 5′-GCCTTCCGTGTCCCCACTG-3′, Reverse: 5′-CGCCTGCTTCACCACCTTC-3′).

### Cytokine measurement

Cytokine levels in cell culture supernatants and in mouse serum were measured by enzyme-linked immunosorbent assay (ELISA). The ELISA kits for IFN-γ, IL-6, TNF-α, and IL-12p40 were purchased from eBioscience.

### Bacterial infection *in vitro*

*E.coli* strain O111:B4 and *Salmonella typhimurium* strain SL1344 used in this study were grown in Luria Broth (LB) at 37°C. *L. monocytogenes* ATCC strain 10403S was a gift from Lie Wang (Institute of Immunology, Zhejiang University School of Medicine, China) and was grown in Bacto brain heart infusion (BHI) broth at 37°C. For bacterial infection experiments *in vitro*, bacteria were heat-killed (HK) by incubation at 70°C for 3 h, pelleted by centrifugation, washed with PBS, and resuspended in PBS. Target cells were cultured in 12-well plates and washed with PBS before adding bacteria at different multiplicity of infection (MOI) for the indicated times.

### Survival analysis and bacterial burden assay of *in vivo* infection of *L. monocytogenes*

*L. monocytogenes* was cultured in Bacto BHI broth at 37°C to mid-logarithmic phase, pelleted by centrifugation, washed with PBS, and resuspended in PBS. Mice (8-12 weeks) were injected intravenously with 2×10^5^ colony-forming units (CFUs) of bacteria in 200 μl PBS. For the survival study, mice were observed every day. Survival curves were generated using GraphPad Prism software, and statistical significance was assessed using the log-rank test.

For bacterial burden assay, liver and spleen were collected and homogenized in 5 mL PBS at 1 or 3 days postinfection. Bacterial counts were obtained by plating serial dilutions of each homogenate on BHI agar plates and cultured at 37°C. Data are expressed as the mean CFUs/organ(log_10_)±SEM.

### Liver and spleen histology

The large lobe of the liver and the entire spleen were removed from the mice 3 days postinfection, rinsed in PBS, and placed in 10% buffered formalin. The liver and spleen were dehydrated with increasing concentrations of ethanol, embedded in paraffin, cut into 5-μm sections, and stained with hematoxylin and eosin (H&E). The brightfield images were taken using DP2-BSW application software and an OLYMPUS Microscope (IX71). Two images of different sections of the organs were obtained per mouse.

### Phagocytosis and bacteria-killing assay

Phagocytosis was assessed by flow cytometry. Cells were infected with Alexa Fluor 488-labeled heat-killed *L. monocytogenes* at a MOI of 10 for the indicated times at 37°C. Then, the cells were extensively washed with cold PBS twice and fixed with 4% paraformaldehyde. The fluorescence of the extracellular bacteria was quenched by replacement of the medium with 0.2% Trypan blue in PBS, pH 5.5, shortly before the actual measurement by flow cytometry. Data are expressed as mean fluorescence intensity (MFI) of total cells.

Bacterial killing was assessed by a gentamicin protection assay [36]. Fresh overnight cultures of *L. monocytogenes* were suspended in PBS and were opsonized with fresh mouse serum used to infect macrophages in medium without antibiotics. After 30 min at 37°C, gentamicin (50 μg/mL) was added, and the cells incubated for another 30 min at 37°C, washed, and incubated in 1 mL fresh medium at 37°C. Cells were lysed at 0, 1.5, 3, 6 h in 1 mL water for 5-10 min. Serial dilutions were plated on BHI plates containing chloramphenicol, and colonies were counted the next day to determine CFUs. Data are expressed as the mean±SEM.

### Phagosomal pH measurement of macrophages infected with *L. monocytogenes*

Phagosomal pH was determined by dual fluorescence flow cytometry [37]. Heat-killed *L. monocytogenes* was covalently coupled with FITC (pH sensitive) and Alexa Fluor 647 (pH insensitive) in the presence of sodium hydrogen carbonate buffer at pH 8 for 2 h at room temperature. After extensive washing with 100 mM glycine, *L. monocytogenes* cells were suspended in PBS for use. The cells were pulsed with the coupled *L. monocytogenes* (MOI, 10) for 20 min and then extensively washed in cold PBS. The cells were incubated at 37°C (‘‘chased’’) for the indicated times and immediately analyzed by FACS. The ratio of the MFI emission between the two dyes was determined. Values were compared with a standard curve obtained by resuspending the cells that had phagocytosed *L. monocytogenes* for 2 h at a fixed pH (ranging from pH 4.5 to 8) and containing 0.1% Triton X-100. The cells were immediately analyzed by FACS to determine the emission ratio of the two fluorescent probes at each pH.

### Immunoprecipitation and immunoblot analysis

Cells were lysed in NP-40 lysis buffer containing 50 mM Tris (pH 7.4), 150 mM NaCl, 1% NP-40, 10 mM phenylmethylsulfonyl fluoride (PMSF), 20 mM NaF, 1 mM Na_3_VO_4_ and a protease inhibitor ‘cocktail’ (Sigma). Lysates were immunoprecipitated with the appropriate antibodies for 4 h at 4°C. Protein A/G Sepharose beads (Roche) were then added, and the samples were incubated overnight. Alternatively, lysates were immunoprecipitated with anti-Flag M2 Magnetic beads (Sigma) for 4 hours at 4°C. After three washes, samples were resolved on SDS-PAGE gels (10% or 12%) and blotted. For immunoblot analysis, cells were lysed in SDS sample buffer by the addition of 1/4 volume of 5×SDS sample buffer directly into the cell suspensions. Samples were then boiled for 5 min and separated using 10% or 12% SDS-PAGE.

### Immunofluorescence and confocal microscopy

Cells were fixed in prewarmed 4% paraformaldehyde for 30 min and permeabilized with 0.2% Triton X-100 for 10 min. After the samples were blocked with 5% BSA, cells were incubated overnight with antibody (1:50 in PBS containing 5% BSA). Staining was detected using DyLight 488- or DyLight 549-labeled secondary antibody (Multiscience). Nuclei were co-stained with DAPI (Roche). Stained cells were imaged using a confocal fluorescence microscope (IX81-FV1000; Olympus). The percentage of *L. monocytogenes* or Flag-SNX10 that co-localized with Rab5, Rab7 or LAMP1 was quantitatively analyzed using the co-localization dialogue of Metamorph offline software by randomly scanning > 20 cells in each test group in two or more independent experiments [38].

### Statistical analysis

All statistical analyses were performed using GraphPad Prism software. Statistical significance was determined by Student's *t*-test (two-tailed distribution with a two sample equal variance). Survival curves were generated using GraphPad Prism software, and the log-rank test was used to assess statistical significance among groups of mice, with *p* values < 0.05 considered significant.

## SUPPLEMENTARY MATERIALS FIGURES


