# Supplementary material for: SNX10 promotes phagosome maturation in macrophages and protects mice against Listeria monocytogenes infection
Source: Oncotarget. 2017 Jul 27;8(33):53935–47. doi: 10.18632/oncotarget.19644 (PMC5589552; doi:10.18632/oncotarget.19644)
Supplement: Supplementary file 1 [file oncotarget-08-53935-s001.pdf]

SNX10 promotes phagosome maturation in macrophages and protects mice against *Listeria monocytogenes* infection

SUPPLEMENTARY MATERIALS

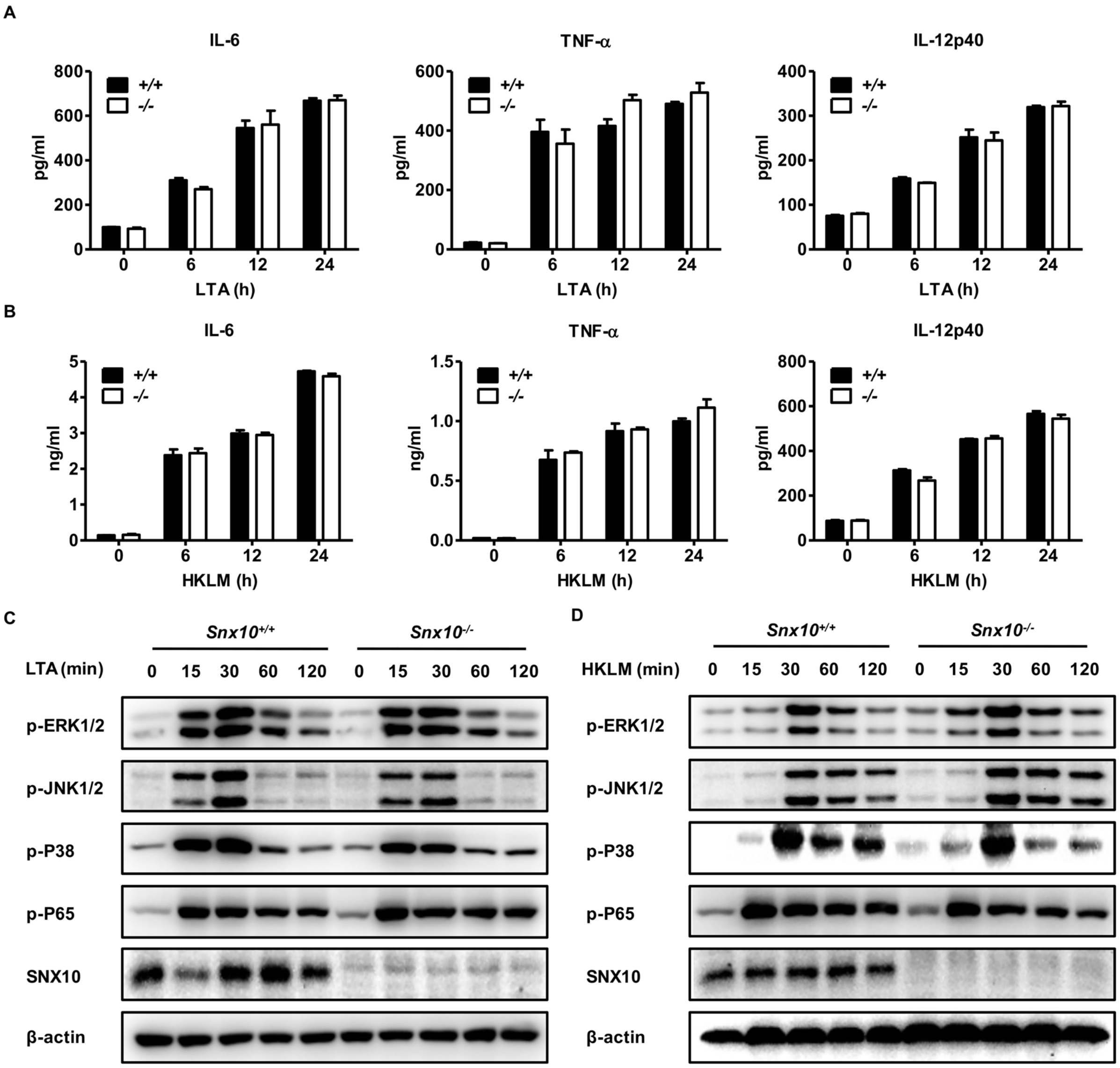

Supplementary Figure 1: Loss of SNX10 does not affect TLR signaling pathways and cytokine production. (**A**, **B**) ELISA of TNF-α, IL-6 and IL-12p40 in the supernatant of WT or *Snx10*$^{-/-}$ BMDMs after LTA (2 µg/mL) stimulation or HKLM (MOI, 20) infection for the indicated time. (**C**, **D**) Immunoblot analysis of phosphorylated (p-) ERK, JNK, P38 and P65 in lysates of WT or *Snx10*$^{-/-}$ BMDMs after LTA (2 µg/mL) stimulation or HKLM (MOI, 20) infection for the indicated time, with β-actin as a loading control. Data are representative of three independent experiments with similar results.

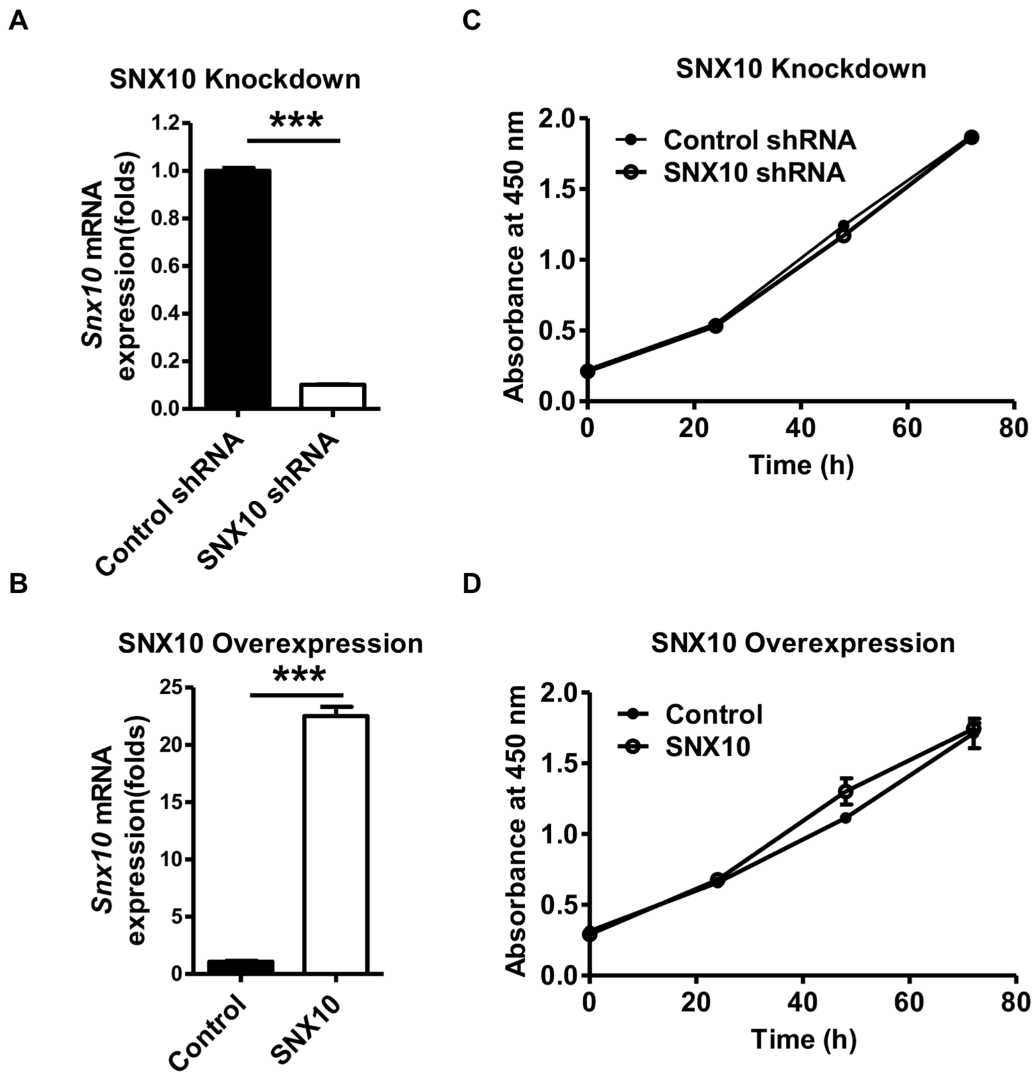

Supplementary Figure 2: SNX10 does not affect the proliferation of macrophage. (**A**, **B**) Detection of SNX10 level in RAW264.7 cells stably expressing control and SNX10 shRNA (A), or stably expressing control and SNX10 (B) by Q-PCR analysis, ***P<0.001. (**C**, **D**) Cell proliferation assay of RAW264.7 cells stably expressing control and SNX10 shRNA (C), or stably expressing control and SNX10 (D) using CCK-8, Data are expressed as the absorbance at 450 nm. Data are representative of three independent experiments with similar results.

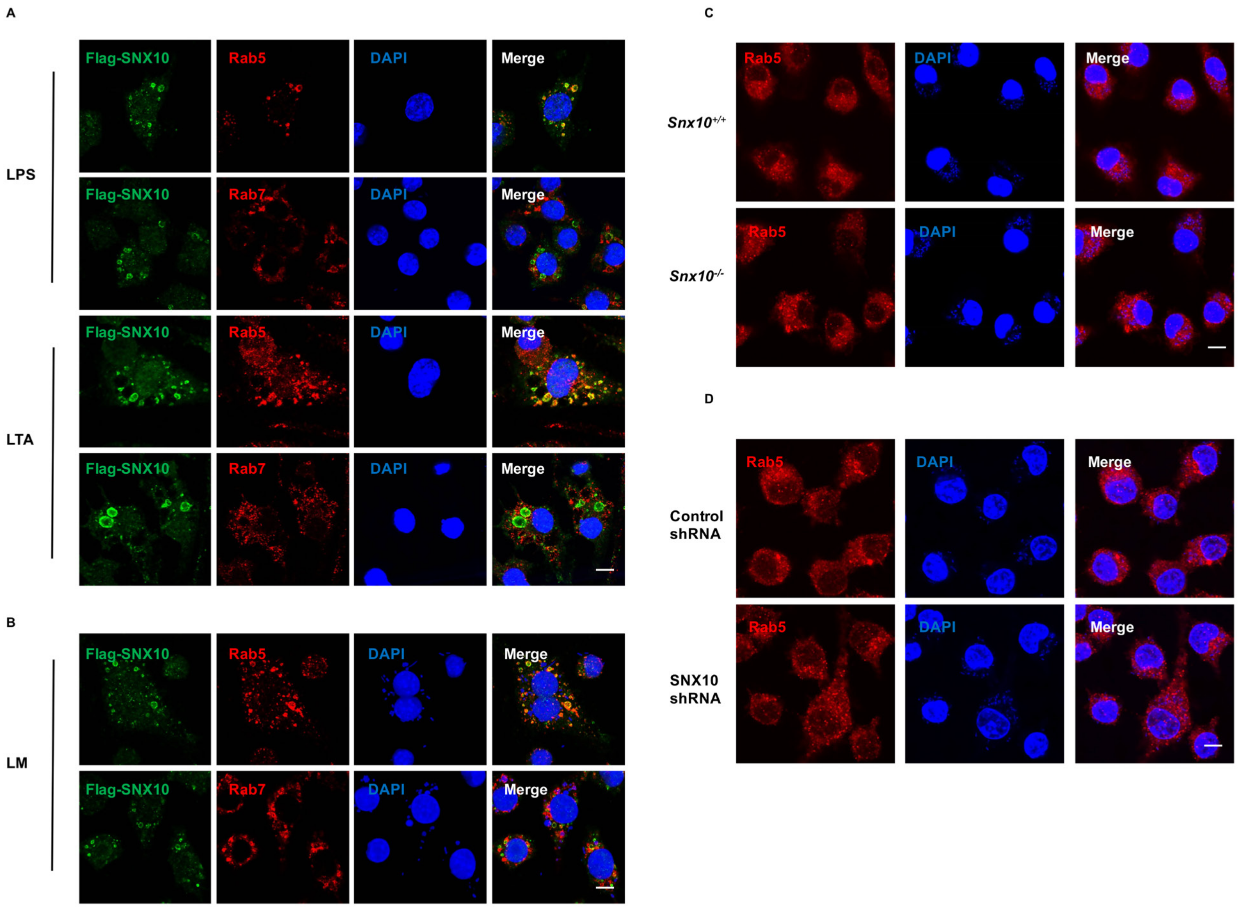

Supplementary Figure 3: Absence of SNX10 does not affect early endosomes and phagosomes. (**A**, **B**) Confocal images showing the co-localization of Flag-SNX10 (Green) with Rab5, Rab7 (Red) in RAW264.7 cells stably expressing Flag-SNX10 under LPS (1 μg/mL) or LTA stimulation (2 μg/mL) (A) or *L. monocytogenes* (LM) infection (MOI, 20) (B) for 3 h. Co-localzation of the two colors is depicted by yellow in the images. (**C**, **D**) Confocal images showing the early endosomes or phagosomes (Rab5-positive) in WT and *Snx10*$^{-/-}$ BMDMs (C) or RAW264.7 cells stably expressing control or SNX10 shRNA (D) after infection with *L. monocytogenes* (MOI, 20) for 3 h. Scale bars represent 10 μm. Data are representative of three independent experiments with similar results.

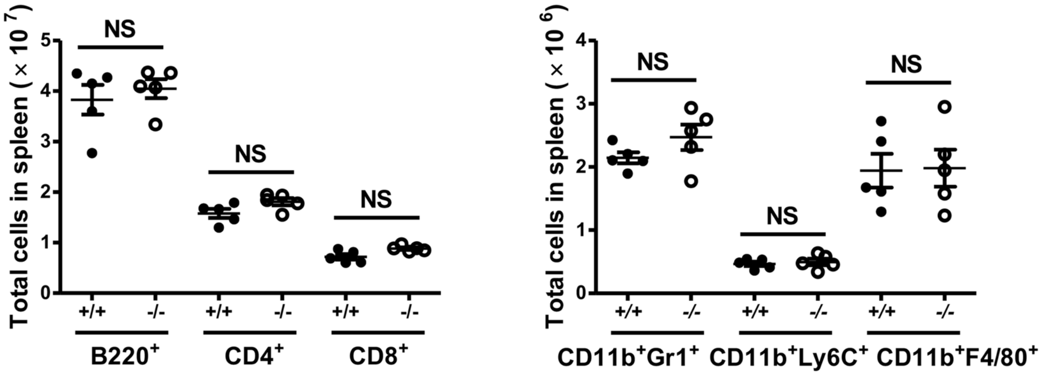

Supplementary Figure 4: There are similar numbers of immune cells in WT and Snx10$^{-/-}$ mice after injection with PBS. Different cell types were counted in WT and *Snx10*$^{-/-}$ mice after intravenous injection with PBS for 72 h. NS, no significance. Data are representative of three independent experiments with similar results.

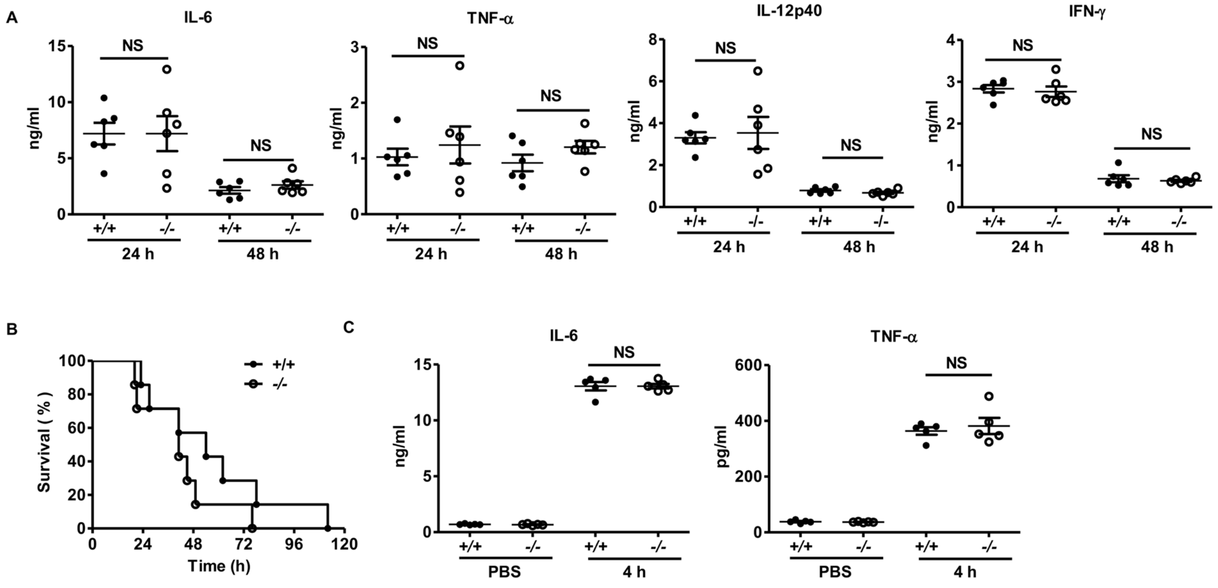

Supplementary Figure 5: SNX10 deficiency has no effect on the production of cytokines after infection *in vivo*. (**A**) ELISA of TNF-α, IL-6, IFN-γ, and IL12p40 in serum collected at 24 or 48 h after intravenous injection with 2×10^5 *L. monocytogenes*; (**B**) Survival of WT and *Snx10*$^{-/-}$ mice after intraperitoneal injection with LPS (15 mg/kg body weight) and monitored every hour after infection, n=7 mice per genotype; (**C**) ELISA of TNF-α and IL-6 in serum collected at 4 h after intraperitoneal injection with PBS or LPS as B, each symbol represents an individual mouse. NS, no significance. Data are representative of three independent experiments with similar results.
